# Usability and Acceptability of a Palliative Care Mobile Intervention for Older Adults With Heart Failure and Caregivers: Observational Study

**DOI:** 10.2196/35592

**Published:** 2022-10-06

**Authors:** Jennifer Paola Villalobos, Sheana Salyers Bull, Jennifer Dickman Portz

**Affiliations:** 1 Colorado School of Public Health University of Colorado Aurora, CO United States; 2 Division of General Internal Medicine University of Colorado Aurora, CO United States

**Keywords:** mHealth, older adult, symptom, heart failure, palliative care, app, digital health, cardiology, heart, Convoy-Pal, mobile, tablet, smartwatch, adult, aging

## Abstract

**Background:**

Heart failure is a leading cause of death among older adults. Digital health can increase access to and awareness of palliative care for patients with advanced heart failure and their caregivers. However, few palliative care digital interventions target heart failure or patients’ caregivers, family, and friends, termed here as the social convoy. To address this need, the Social Convoy Palliative Care (Convoy-Pal) mobile intervention was developed to deliver self-management tools and palliative care resources to older adults with advanced heart failure and their social convoys.

**Objective:**

The goal of the research was to test the acceptability and usability of Convoy-Pal among older adults with advanced heart failure and their social convoys.

**Methods:**

Convoy-Pal includes tablet-based and smartwatch tools facilitating self-management and access to palliative care resources. Older adults and social convoy caregivers completed an acceptability and usability interview via Zoom, including open-ended questions and the Mobile Application Rating Scale: User Version (uMARS). Descriptive analysis was conducted to summarize the results of open-ended feedback and self-reported acceptability and usability.

**Results:**

A total of 26 participants (16 older adults and 10 social convoy caregivers) participated in the interview. Overall, the feedback from users was good (uMARS mean 3.96/5 [SD 0.81]). Both older adults and social convoy caregivers scored information provided by Convoy-Pal the highest (mean 4.22 [SD 0.75] and mean 4.21 [SD 0.64], respectively). Aesthetics, functionality, and engagement were also perceived as acceptable (mean >3.5). Open-ended feedback resulted in 5 themes including improvements to goal setting, monitoring tools, daily check-in call feature, portal and mobile app, and convoy assessment.

**Conclusions:**

Convoy-Pal was perceived as acceptable with good usability among older adults with heart failure and their social convoy caregivers. With good acceptability, Convoy-Pal may ultimately lead to increased access to palliative care resources and facilitate self-management among older adults with heart failure and their social convoy caregivers.

## Introduction

Heart failure (HF) is the 4th leading cause of death from heart disease in the United States and is most prevalent among individuals aged 65 years and older (ie, older adults). According to data from 2015 to 2018, 7.5% of males and 3.9% of females aged 60 to 79 years have HF [1]. The prevalence of HF continues to rise over time as the population ages [2]. By the year 2040, the number of older Americans is expected to nearly double to an estimated 80.8 million [3]. As the prevalence of HF increases, the need for palliative care amplifies. Palliative care can be beneficial for patients with HF as well as their caregivers, families, friends, and loved ones, referred to here as the social convoy [4]. Palliative care offers a support system to help the social convoy cope during the patient’s illness and effectively control distressing symptoms experienced by patients with HF [5]. In general, symptom control and good communication are basic palliative care principles highly recommended to improve the quality of life for patients with HF [5]. Although relatively underexplored, digital health [6] innovations (ie, telehealth, wearable devices, and mobile health [mHealth]) provide modern opportunities for patients and their social convoy to engage in palliative care [7-11].

Although there is a need, few studies focus on HF-specific mHealth in palliative care or mHealth supports for the social convoy. A systematic review of mHealth in palliative care reports that the primary uses of mobile apps are for biological and clinical monitoring (75% of the apps), disease self-management (64% of the apps), and therapeutic patient education (50% of the apps) [12]. One pilot in the review targets patients with HF and has found that the use of the HF mobile app improves self-care management [13]. Another study involving HF patients and their informal caregivers shows mHealth may decrease risk of HF exacerbations and improve caregiver communication [14]. While there are early indicators that patients and caregivers benefit from mHealth, providers also express enthusiasm about the potential of mHealth in palliative care [15-17]. Palliative care providers recommend digital health innovations in the areas of telehealth, client health records, and personal health tracking [17].

Quality testing in mHealth includes acceptability and usability as standard and essential in the field. Acceptability testing is usually completed first, followed by usability testing. This type of testing, for example, allows researchers to increase confidence that subsequent research on the efficacy of a tool produces outcomes that ensure null or negative outcomes are not due to poor tool function. Essentially, acceptability testing in mHealth assists with determining the level of meaningful engagement with the app; otherwise, if not engaging, the app will not be used, which may affect retention over time [18]. Usability testing, on the other hand, highlights the need to adapt the apps to users’ needs to create more usable tools [19] and ensure an app can be used the way it was intended by the specific audience for the tool [20].

Given the limited access to HF-specific palliative care mHealth, the Social Convoy Palliative Care Mobile Intervention, Convoy-Pal, was developed in response to a need for self-care strategies for both older adults with HF and their social convoys. Convoy-Pal was co-designed with older adults, caregivers, and health care providers [21] under the clinical guidelines establish by the National Coalition for Hospice and Palliative Care [22]. As a step in the co-designing process, this study was to test the acceptability of Convoy-Pal among older adults with HF and their caregivers.

## Methods

### Convoy-Pal Platform

The authors are researchers at the mHealth Impact Lab [23] who contract with Routinify, Inc, [24], a vendor that delivers the Convoy-Pal intervention. Routinify offers a variety of software and hardware tools that are publicly available; costs vary based on the tools provided and can range from US $50 to $100 per patient. In this case, Routinify assists with the delivery of the Convoy-Pal intervention to older adults and their social convoys. However, Routinify is only permitted to deliver Convoy-Pal in contract with the mHealth Lab and is not engaged in the clinical research (ie, they are not involved in the study instruments, data collection, management, analysis, or designing the protocol).

**Figure 1:**
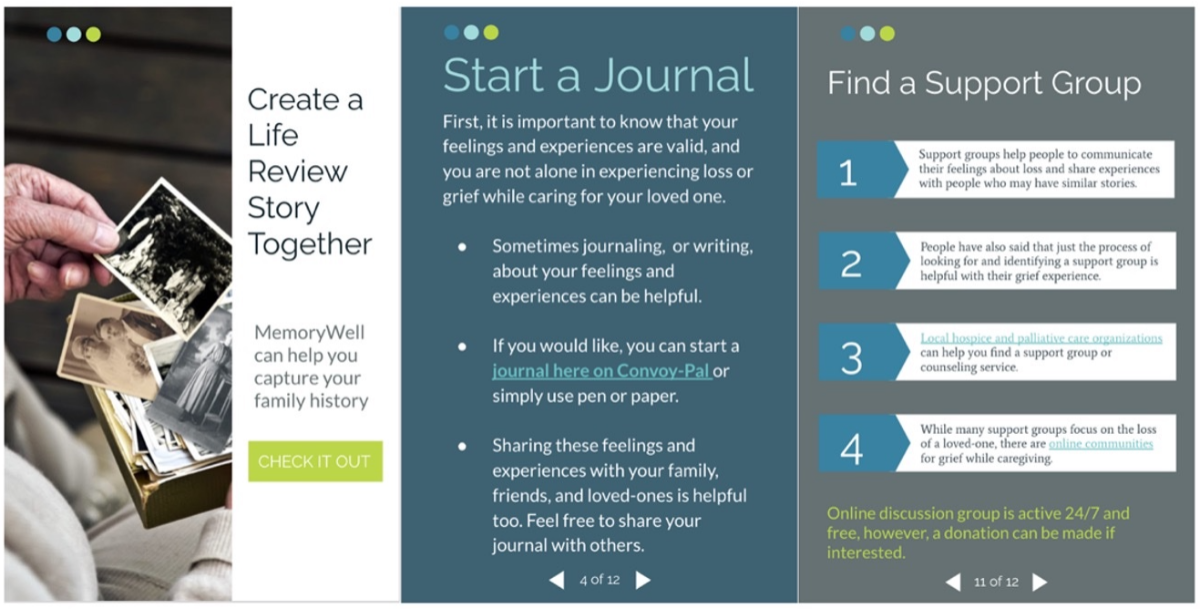
Near end-of-life aspects of care.

Convoy-Pal is designed with the following care domains: physical, psychological, social, spiritual, near end of life, ethical and legal, and knowledge about palliative care overall. Convoy-Pal includes a palliative care assessment with self-monitoring and resource tools for each domain. For example, the near end-of-life aspects of care (Figure 1) includes information regarding grief support and self-care and provides an opportunity for life review activities. This also includes resources on how to communicate unaddressed concerns and identify a support group for social support. Convoy-Pal tools and content are designed to be delivered via WellAssist by Routinify, Inc. WellAssist is a personal point-of-care app and associated internet-connected medical devices. The app’s core is based on behavioral modifications in line with the overall plan of care. The app is designed so that all members within the social convoy can access and use the Convoy-Pal intervention.

### Ethics Approval and Considerations

Study procedures were approved by the Colorado Multiple Institutional Review Board (number 18-0973). All participants electronically consented to participate. Convoy-Pal collects assessments, including smartwatch-captured vital information, regarding mental health and overall well-being data for the older adult patient and caregivers. It is ethically sound to obtain consent from older adults to share their health information with their caregivers as we did for this study; however, older adult patients also have the option not to share their health information with their caregivers if desired.

### Recruitment

Potential patient participants were identified from the UCHealth University of Colorado Hospital health system’s electronic medical record. Potential participants were aged at least 65 years at the time of recruitment and had been hospitalized at the UCHealth Hospital more than 2 times for HF in the year prior (January 2020-2021). Participants were currently living in their homes and receiving follow-up HF care. We mailed a study invitation letter with an opt-out contact option. Patients who did not opt out were then contacted by phone for recruitment and asked to self-identify social convoy caregivers.

### Data Collection

Two research coordinators (JPV and IM) interviewed participants via Zoom to gain feedback on Convoy-Pal. Participants were exposed to sections of Convoy-Pal throughout the interview process, which lasted between 40 minutes to 1 hour. Participants were shown the Convoy-Pal hardware, which consisted of the tablet, watch, and charging station, and the web-based system portal and mobile app during the interview. Participants were also shown Convoy-Pal features such as goal setting and planning, monitoring options, daily check-in and calling features, convoy caregiver assessments, and palliative care resources. During the exposure to Convoy-Pal, participants completed a self-report acceptability measure and were asked to provide open-ended feedback.

For self-report acceptability, participants completed the Mobile Application Rating Scale: User Version (uMARS) survey [25]. The uMARS survey comprises 4 objective quality subscales—(1) engagement with the app, (2) functionality and users’ perceived functioning of the app, (3) aesthetics, and (4) users’ perception of the quality of the information [25]—to determine app quality mean score. uMARS has 2 optional subscales that can be used depending on the aims of the research. These 2 subscales are the app subjective quality scale, which can be reported as individual items, and the perceived impact scale, which obtains information on the knowledge, attitudes, and behavior change toward improving health behavior [25]. All items are assessed on a 5-point scale, with a uMARS score of 5 considered excellent while a score of 1 is inadequate [26]. The uMARS is shown to have high interrater reliability for evaluating the quality of mHealth apps on well-being, for example [25,27].

For open-ended feedback, the tablet and smartwatch were introduced and displayed over Zoom to the participants. We used a semistructured interview guide (Multimedia Appendix 1) to ask the participants questions and their opinions regarding the hardware, goal setting and planning, monitoring options, daily check-in and convoy calling options, portal and mobile app for convoy, convoy assessments, and convoy resources. Notes, recommendations, and opinions from participants were archived into Qualtrics (Qualtrics) [28], data management software, as the interview was being conducted. The final data captured were stored and saved in Qualtrics with their study ID numbers.

### Data Analysis

The uMARS survey data was analyzed using Excel (Microsoft Corp) calculation mechanisms and descriptive frequencies including mean scores for both caregivers and patients. Once all the interviews were complete, the qualitative data were moved from Qualtrics to NVivo 12 (QSR International) [29], a qualitative research software package, for analysis. A preliminary codebook was created, incorporating explicit domains from the interview guide (deductive themes) by a research assistant (JPV). A descriptive qualitative approach [30] was then used to identify themes and subthemes [31,32]. The codebook and final data interpretation were discussed in a group with all authors. No member checking was conducted.

## Results

### Participants

We recruited 26 participants (16 patients and 10 caregivers) from the University of Colorado Denver and its affiliate, the University of Colorado Hospital. Patients and convoy caregivers participated together or separately depending on the patient’s ability to participate in the interview. Patients were primarily males (9/16, 56%), White (14/16, 88%), and had a mean age of 76 (SD 5) years. Caregivers were predominantly female (7/10, 70%), White (7/10, 70%), and had a mean age of 71 (SD 10) years. Patients were married (12/16, 75%) and had a postgraduate degree (8/16, 50%), with 44% (7/16) having an income of US $30,000 or more and 82% (13/16) owning an iPhone, Android, or a regular or basic phone (Table 1). Similarly, most caregivers were married (8/10, 80%) and had a college or postgraduate degree (7/10, 70%), with 50% (5/10) making US $30,000 or more; 40% (4/10) of caregivers chose not to answer the question regarding their income. All of the caregivers owned an iPhone, Android, or a regular basic phone (Table 1). Due to small cell sizes, demographic categories were collapsed and are not reported in the table.

**Table 1:** Participant technology use.

Technology use		Patients, n (%) (n=16)	Caregivers, n (%) (n=10)	Total, n (%) (N=26)
**Cell phone**				
	Basic phone: iPhone, Android, or regular or basic phone	13 (82)	10 (100)	23 (84)
	I do not have a cell phone	1 (<1)	0	1 (<1)
	Did not respond	2 (13)	—^a^	2 (1)
**Digital activity**				
	Email	16 (100)	10 (100)	26 (100)
	Look up information	16 (100)	10 (100)	26 (100)
	Use social media	16 (100)	6 (60)	22 (84)
	Post and share pictures or videos	15 (94)	9 (90)	24 (92)
	Read or post comments	15 (94)	10 (100)	25 (96)
	Play computer games	14 (88)	10 (100)	24 (92)
	Video chat	16 (100)	10 (100)	26 (100)
	Instant message or chat rooms	13 (82)	7 (70)	20 (76)

^a^Not applicable.

### Acceptability

#### Mobile Application Rating Scale: User Version

Overall, the acceptability feedback from users was good. The uMARS mean score was 4.00 (SD 0.78) among patients and 3.92 (SD 0.83) among caregivers, with an overall uMARS mean score of 3.96 (SD 0.81) among both groups (Table 2). Patients and caregivers showed the most concordance with Section D: information scale and the most discordance with Section C: aesthetics (Table 2). Further description of the mean, standard deviation, and minimum and maximum values for the subscales of the uMARS are provided in Multimedia Appendix 2.

Examining uMARS domain scores individually, we found that patients gave Section D: information the highest rating (mean 4.22, SD 0.75), followed by Section C: aesthetics (mean 4.13, SD 0.73), Section B: functionality (mean 3.87, SD 0.85), and Section A: engagement (mean 3.80, SD 0.79). Patients scored the app’s subjective quality scale a mean of 4.01 (SD 0.70) and the perceived impact of the app on the user’s knowledge, attitudes, and intentions related to the target health behavior a 3.64 (SD 0.96). Similarly, caregivers scored Section D: information the highest (mean 4.21, SD 0.64), followed by Section C: aesthetics (mean 3.89, SD 0.72), Section B: functionality (mean 3.82, SD 1.0), and Section A: engagement (mean 3.77, SD 0.96). The app subjective quality scale was rated mean 3.56 (SD 1.23) and perceived impact was rated mean 3.13 (SD 1.20) among caregivers.

**Table 2:** Mean, standard deviation, and range values for the subscales of the uMARS (Mobile Application Rating Scale: User Version).

	Patients			Caregivers	
	Mean (SD)	Range	Mean (SD)		Range
Section A: engagement	3.80 (0.79)	2.25-5.00	3.77 (0.96)		2.40-5.00
Section B: functionality	3.87 (0.85)	2.00-5.00	3.82 (1.00)		2.50-5.00
Section C: aesthetics	4.13 (0.73)	3.33-5.00	3.89 (0.72)		3.00-5.00
Section D: information	4.22 (0.75)	2.50-5.00	4.21 (0.64)		3.50-5.00
Total	4.00 (0.78)	—^a^	3.92 (0.83)		—

^a^Not applicable.

#### Open-Ended Feedback

Five main themes were identified after receiving open-ended feedback: goal setting, monitoring tools, daily check-in call feature, portal and mobile app, and convoy assessment. Representative quotes for themes and additional subthemes are reported in Table 3.

#### Goal Setting

Participants expressed the need for the goal-setting section to provide realistic and obtainable goals. For example, it was expressed that goal setting should be addressed monthly, not weekly. Additionally, participants expressed that they would like an option to add a comment box to include other action items and or commentary for their goals.

**Table 3:** Participant feedback (N=23).

Theme	Subthemes	Representative quote
Goal setting	Obtainable goals	“Questions should be addressed monthly not weekly.” [72-year-old participant]
	Comment section	“Provide fill-in-the-blank options.” [75-year-old participant]
Monitoring tools	Added features	“Would like to see prompting feedback if things are not okay.” [84-year-old participant] “Add ways to detect stroke and falls down the stairs.” [74-year-old participant]
Reminders	Checklist	“Design a checklist of all of the medication a person takes for specific medication notifications versus getting general messages.” [71-year-old participant]
Portal and mobile app	Thresholds	“Would like to see thresholds on the graphs to determine who should be consulted.” [74-year-old participant]
Convoy assessment	Wrong approach	“if [a caregiver] is in crisis mode, they will not fill out the questions...this is not beneficial for patients who need extra help and support.” [72-year-old participant] “They would not answer those questionnaires truthfully because they were not raised to share emotions growing up.” [78-year-old participant]

#### Monitoring Tools

Participants agreed that the monitoring tools were helpful for people with HF and other chronic conditions. Feedback to improve the monitoring tools included the addition of other features, such as feedback prompting concerning vitals, electrocardiogram measures, fall detection, stroke indicators, and heart palpitation monitoring.

#### Reminder and Call Feature

Most of the participants liked the daily check-in and call feature. One participant said “...the feature is good for people who live alone and want to keep in contact via FaceTime with their loved ones” (72-year-old participant). A few participants who disliked the daily check-in feature expressed that some patients might feel burdened by the frequency of check-ins. Others indicated the feature was redundant as they could schedule reminders and calls through their personal phone instead. One suggestion included designing a checklist of all of the medication a person takes and getting notifications on those specific mediations versus just getting a general message.

#### Portal and Mobile App

The majority of the participants liked the portal and mobile app. The participants appreciated that the portal, charts, and layout of the mobile app were clear and concise. Participants also liked the opportunity to share access to personal data with family members. Feedback from 2 participants included adding thresholds to the graphs to determine who, such as a provider or family member, should be consulted, and adding instructions on who to call with concerns.

#### Social Convoy Assessment

Participants provided many recommendations when asked their opinions about convoy assessment. Many participants were hesitant about the caregiver assessments due to time, burden, and specific assessment topics. For example, in the domain of emotional assessment, a participant said “if [a caregiver] is [in] crisis mode they will not fill out the questions...this is not beneficial for patients who need extra help and support” (Table 3). Another participant expressed that “they would not answer those questionnaires truthfully because they were not raised to share emotions growing up” (Table 3). Additionally, a participant expressed that some assessments should be addressed in person and not via the tablet. Other themes that arose from the feedback included ensuring assessments are HIPAA (Health Insurance Portability and Accountability Act) compliant and appropriate language is used. For example, one participant responded, “The ‘I feel sad’ language might not be appropriate for people because they are not readily going to admit that they are sad” (71-year-old participant). Participants also suggested that the assessments should not take too long to complete.

## Discussion

### Principal Findings

Convoy-Pal is designed to increase access to palliative care resources and self-management in the setting of HF. Acceptability testing is essential because it results in a better quality product. In this acceptability and usability study, the Convoy-Pal is considered acceptable and a good quality app, based on the uMARS scores among older adults with HF and their caregivers. Older adult patients and caregivers also provided recommendations for improving Convoy-Pal, which included adding comment sections, designing a checklist for medications, including thresholds on graphs for interpretation, and adding features such as fall detection. Based on this feedback, authors will update and continue to assess Convoy-Pal for usability and feasibility.

Although there are few high-quality HF mobile apps [33] assessed for acceptability, functionality, and efficacy [33,34], our findings are supported by other empirical studies. For example, palliative care patients found a mobile mortality risk tool acceptable to use [35]. Additionally, using wearables for monitoring palliative care was also feasible [36,37], a tool that Convoy-Pal offers with its smartwatch. Similar to the feedback provided for Convoy-Pal, a commentary article, systematic meta-review, and qualitative study [15,38,39] report the need to track relevant information, receive education pertinent to health for older adults, and provide information sharing such as medication use.

Aside from HF apps specifically, digital health interventions overall have the potential to improve the accessibility and effectiveness in palliative care, as reported by a recent systematic meta-review [38]. Palliative care is one area where technologies are increasingly being deployed. Although leveraging existing resources for palliative care is one approach, mHealth interventions targeting palliative care enable patients increased access to this resource without spending time or traveling to locations [40]. mHealth palliative care allows older adults to participate in and govern their care. For example, they do this by self-reporting symptoms and needs, which improves communication with providers and caregivers [38,41,42]. Traditional palliative care resources do not provide a self-governing element in this unique way.

Additionally, HF mobile interventions rarely target the social convoy or palliative care domains. Therefore, Convoy-Pal would contribute to the advancement of palliative care and HF mHealth while also advancing a team approach to information sharing and targeting family- and caregiver-specific issues [43]. Convoy-Pal has the potential to support older adults with HF and their social convoy in the management of physical, psychosocial, and spiritual concerns.

### Limitations

First, due to university and state COVID-19 restrictions, research assistants were not able to meet with older adult and caregiver participants to physically interact with Convoy-Pal on the Routinify tablets or complete assessments in person. Researchers therefore collected acceptability and usability data by displaying Convoy-Pal and all its features remotely to participants for about 1 hour through Zoom. Second, the uMARS survey, for this reason, was modified by our team to reflect the following 2 optional responses for all subscales of the survey: (1) “Optional: Missing due to lack of time with app” and (2) “Optional: Did not feel comfortable answering.” If the participant did not feel comfortable answering the uMARS questions due to their belief that there was not enough time to explore Convoy-Pal, then they could select either optional response. The minor modifications made to the uMARS had not previously been tested and therefore may have reduced the validity of the original items. Physical interaction with the hardware may have yielded additional user feedback. Last, the study was further limited by small sample size and a single health system as well as lack of diversity representative of the local community. Acceptability and usability of Convoy-Pal may differ in other regional areas and varying access to health care.

### Conclusion

HF is a leading cause of death in the United States, and mHealth provides opportunities for patients and their social convoy to participate in palliative care. In our study, 16 older patients and 10 caregivers were interviewed and asked to complete a uMARS assessment and provide open-ended feedback. Overall, older patients and their caregivers perceived Convoy-Pal as acceptable with good usability. Although in-person usability testing is needed, Convoy-Pal was perceived acceptable and may ultimately increase access to palliative care resources and facilitate self-management among older adults with HF and their caregivers.

## Appendix

Multimedia Appendix 1 Convoy-Pal Acceptability Interview Guide.

Multimedia Appendix 2 Mean, standard deviation, and minimum and maximum values for the scales and subscales of the uMARS (Mobile Application Rating Scale: User Version).

## Abbreviations

Convoy-Pal: Social Convoy Palliative Care
HF: heart failure
mHealth: mobile health
uMARS: Mobile Application Rating Scale: User Version

## Data Availability

The data sets generated and analyzed during this study are not yet publicly available. The full study protocol and data sets will be shared to a public data repository upon the conclusion of the Convoy-Pal feasibility study.

## References

[1] (2021). Heart and Stroke Statistics. American Heart Association.

[2] Virani SS, Alonso A, Aparicio HJ, Benjamin EJ, Bittencourt MS, Callaway CW, Carson AP, Chamberlain AM, Cheng S, Delling FN, Elkind MSV, Evenson KR, Ferguson JF, Gupta DK, Khan SS, Kissela BM, Knutson KL, Lee CD, Lewis TT, Liu J, Loop MS, Lutsey PL, Ma J, Mackey J, Martin SS, Matchar DB, Mussolino ME, Navaneethan SD, Perak AM, Roth GA, Samad Z, Satou GM, Schroeder EB, Shah SH, Shay CM, Stokes A, VanWagner LB, Wang N, Tsao CW, American Heart Association Council on EpidemiologyPrevention Statistics CommitteeStroke Statistics Subcommittee (2021). Heart Disease and Stroke Statistics-2021 Update: A Report From the American Heart Association. Circulation.

[3] (2021). 2020 Profile of Older Americans. Administration for Community Living.

[4] Antonucci TC, Ajrouch KJ, Birditt KS (2014). The convoy model: explaining social relations from a multidisciplinary perspective. Gerontologist.

[5] Ward C (2002). The need for palliative care in the management of heart failure. Heart.

[6] (2022). Digital Health Center of Excellence. US Food and Drug Administration.

[7] Capurro D, Ganzinger M, Perez-Lu J, Knaup P (2014). Effectiveness of eHealth interventions and information needs in palliative care: a systematic literature review. J Med Internet Res.

[8] Head BA, Schapmire TJ, Zheng Y (2017). Telehealth in Palliative Care. J Hosp Palliat Nurs.

[9] Tasneem S, Kim A, Bagheri A, Lebret J (2019). Telemedicine Video Visits for patients receiving palliative care: A qualitative study. Am J Hosp Palliat Care.

[10] Weck CE, Lex KM, Lorenzl S (2019). Telemedicine in Palliative Care: Implementation of New Technologies to Overcome Structural Challenges in the Care of Neurological Patients. Front Neurol.

[11] Worster B, Swartz K (2017). Telemedicine and Palliative Care: an Increasing Role in Supportive Oncology. Curr Oncol Rep.

[12] Bienfait F, Petit M, Pardenaud R, Guineberteau C, Pignon A (2020). Applying M-Health to Palliative Care: A Systematic Review on the Use of M-Health in Monitoring Patients With Chronic Diseases and its Transposition in Palliative Care. Am J Hosp Palliat Care.

[13] Athilingam P, Jenkins B, Johansson M, Labrador M (2017). A Mobile Health Intervention to Improve Self-Care in Patients With Heart Failure: Pilot Randomized Control Trial. JMIR Cardio.

[14] Piette JD, Striplin D, Marinec N, Chen J, Trivedi RB, Aron DC, Fisher L, Aikens JE (2015). A Mobile Health Intervention Supporting Heart Failure Patients and Their Informal Caregivers: A Randomized Comparative Effectiveness Trial. J Med Internet Res.

[15] Son Y, Oh S, Kim EY (2020). Patients' needs and perspectives for using mobile phone interventions to improve heart failure self-care: A qualitative study. J Adv Nurs.

[16] Dickman Portz J, Ford K, Bekelman DB, Boxer RS, Kutner JS, Czaja S, Elsbernd K, Bull S (2020). "We're Taking Something So Human and Trying to Digitize": Provider Recommendations for mHealth in Palliative Care. J Palliat Med.

[17] Mills J, Fox J, Damarell R, Tieman J, Yates P (2021). Palliative care providers' use of digital health and perspectives on technological innovation: a national study. BMC Palliat Care.

[18] Materia FT, Smyth JM (2021). Acceptability of Intervention Design Factors in mHealth Intervention Research: Experimental Factorial Study. JMIR Mhealth Uhealth.

[19] Zapata BC, Fernández-Alemán José Luis, Idri A, Toval A (2015). Empirical studies on usability of mHealth apps: a systematic literature review. J Med Syst.

[20] Schnall R, Cho H, Liu J (2018). Health Information Technology Usability Evaluation Scale (Health-ITUES) for Usability Assessment of Mobile Health Technology: Validation Study. JMIR Mhealth Uhealth.

[21] Portz JD, Ford KL, Doyon K, Bekelman DB, Boxer RS, Kutner JS, Czaja S, Bull S (2020). Using Grounded Theory to Inform the Human-Centered Design of Digital Health in Geriatric Palliative Care. J Pain Symptom Manage.

[22] (2018). Clinical Practice Guidelines for Quality Palliative Care, 4th edition. National Coalition for Hospice and Palliative Care.

[23] mHealth Impact Lab. Colorado School of Public Health.

[24] Routinify: Smarter Living.

[25] Stoyanov SR, Hides L, Kavanagh DJ, Wilson H (2016). Development and Validation of the User Version of the Mobile Application Rating Scale (uMARS). JMIR Mhealth Uhealth.

[26] Terhorst Y, Philippi P, Sander LB, Schultchen D, Paganini S, Bardus M, Santo K, Knitza J, Machado GC, Schoeppe S, Bauereiß Natalie, Portenhauser A, Domhardt M, Walter B, Krusche M, Baumeister H, Messner E (2020). Validation of the Mobile Application Rating Scale (MARS). PLoS One.

[27] Stoyanov SR, Hides L, Kavanagh DJ, Zelenko O, Tjondronegoro D, Mani M (2015). Mobile app rating scale: a new tool for assessing the quality of health mobile apps. JMIR Mhealth Uhealth.

[28] (2005). Qualtrics XM.

[29] NVivo.

[30] Padgett D (2016). Qualitative Methods in Social Work Research.

[31] Tashakkori A, Johnson R, Teddlie C, Fargotstein L (2021). Foundations of Mixed Methods Research: Integrating Quantitative and Qualitative Approaches in the Social and Behavioral Sciences, 2nd Ed.

[32] Saldana J, Seaman J (2016). The Coding Manual for Qualitative Researchers, 3 ed.

[33] Masterson Creber Ruth M, Maurer MS, Reading M, Hiraldo G, Hickey KT, Iribarren S (2016). Review and Analysis of Existing Mobile Phone Apps to Support Heart Failure Symptom Monitoring and Self-Care Management Using the Mobile Application Rating Scale (MARS). JMIR Mhealth Uhealth.

[34] Cajita MI, Gleason KT, Han H (2016). A Systematic Review of mHealth-Based Heart Failure Interventions. J Cardiovasc Nurs.

[35] Saunders S, Downar J, Subramaniam S, Embuldeniya G, van Walraven C, Wegier P (2021). mHOMR: the acceptability of an automated mortality prediction model for timely identification of patients for palliative care. BMJ Qual Saf.

[36] Pavic M, Klaas V, Theile G, Kraft J, Tröster Gerhard, Blum D, Guckenberger M (2020). Mobile Health Technologies for Continuous Monitoring of Cancer Patients in Palliative Care Aiming to Predict Health Status Deterioration: A Feasibility Study. J Palliat Med.

[37] Pavic M, Klaas V, Theile G, Kraft J, Tröster Gerhard, Guckenberger M (2020). Feasibility and Usability Aspects of Continuous Remote Monitoring of Health Status in Palliative Cancer Patients Using Wearables. Oncology.

[38] Finucane AM, O'Donnell H, Lugton J, Gibson-Watt T, Swenson C, Pagliari C (2021). Digital health interventions in palliative care: a systematic meta-review. NPJ Digit Med.

[39] Phongtankuel V, Adelman RD, Reid MC (2018). Mobile health technology and home hospice care: promise and pitfalls. Prog Palliat Care.

[40] Widberg C, Wiklund B, Klarare A (2020). Patients' experiences of eHealth in palliative care: an integrative review. BMC Palliat Care.

[41] Guo Q, Cann B, McClement S, Thompson G, Chochinov HM (2017). Keep in Touch (KIT): feasibility of using internet-based communication and information technology in palliative care. BMC Palliat Care.

[42] Pinto S, Almeida F, Caldeira S, Martins JC (2017). The Comfort app prototype: introducing a web-based application for monitoring comfort in palliative care. Int J Palliat Nurs.

[43] Portz JD, Elsbernd K, Plys E, Ford KL, Zhang X, Gore MO, Moore SL, Zhou S, Bull S (2020). Elements of social convoy theory in mobile health for palliative care: scoping review. JMIR Mhealth Uhealth.

