# Supplementary material for: Usability and Acceptability of a Palliative Care Mobile Intervention for Older Adults With Heart Failure and Caregivers: Observational Study
Source: JMIR Aging. 2022 Oct 6;5(4):e35592. doi: 10.2196/35592 (PMC9585449; doi:10.2196/35592)
Supplement: Multimedia Appendix 1 [file aging_v5i4e35592_app1.docx]

ConvoyPal Acceptability Interview Guide

Start of Block: Default Question Block

Q35 Date:

________________________________________________________________

Q37 Study ID Number:

________________________________________________________________

Q36 Provider, Caregiver, or Patient?

________________________________________________________________

Q2 **Overview**
We are first going to spend about 10-15 minutes going through all the Convoy-Pal tools and features. We will ask you to think out load as we show you examples. After reviewing all of the features we will have specific questions for you about what you think about Convoy-Pal.

Q3 Wireframe: Social Convoy **Section 1. The Convoy**I would like to start today by talking about the Social Convoy. We named the tool after this concept. It is a term used to describe a system of social support. So you can see there is a person in the middle that may need extra support to promote health. There are then the people in the nearest circle who closely help the person in the middle. These people can be spouses, professional or informal caregivers, close friend or other family members. The outer circle is the group of people who provide support but not as much as those closest to the center. Maybe friends, grandchildren, neighbors or other types of family.

Q1 Question: Who are some of the people that might be in your Convoy? Probes: Who do you spend time with? What do you like to do and who do you do these activities with?

________________________________________________________________

________________________________________________________________

________________________________________________________________

________________________________________________________________

________________________________________________________________

Q4 Wireframe: WellAssist Components **Section 2. Review the Hardware**We created Convoy-Pal to support everyone in the Convoy. Let’s start off by looking at the hardware used in Convoy-Pal. Hardware includes devices you and your convoy can use to promote health. The devices include a tablet and watch that are provided by us. Other tools include a website and mobile application that members of the Convoy can access on their own computers and smart phones.

Q5 To start using Convoy-Pal, we will show you the tablet and watch. **(On Zoom camera)**Here is the tablet. When someone receives it, they would simply plug in the tablet and turn it on. The tablet sits on this stand. The stand includes a high-grade speaker that is easier to hear. The tablet can charge on the stand, stay on the stand, or move around with you. The stand also includes these USB plugs which allow you to charge your watch.  
 Here is the watch. Again, it will connect and charge with your tablet. **(If needed, explain that Routinify offers many sensor options, but right now Convoy-Pal only uses the watch. In the future, we may add others)**. You would simply put on the watch.
  **(Side Note: We will ask about website and mobile application tools later)**
 **On Zoom: Display the hardware—The tablet; The stand plus speaker; The USB plugs for watch (and other sensors); The watch**

Q6 Questions:
Do you think you could turn on the tablet and watch?

________________________________________________________________

________________________________________________________________

________________________________________________________________

________________________________________________________________

________________________________________________________________

Q7 Would you like instructions for turning on the tablet and watch?

________________________________________________________________

________________________________________________________________

________________________________________________________________

________________________________________________________________

________________________________________________________________

Q8 How would you like these instructions? **Multiple choice** ·      Hardcopy booklet ·      Videos ·      Telephone or Zoom support?

________________________________________________________________

________________________________________________________________

________________________________________________________________

________________________________________________________________

________________________________________________________________

Q9 Before talking about what the tablet and watch can do, are there things you like about the tablet and watch?

________________________________________________________________

________________________________________________________________

________________________________________________________________

________________________________________________________________

________________________________________________________________

Q10 Are there things you do not like?

________________________________________________________________

________________________________________________________________

________________________________________________________________

________________________________________________________________

________________________________________________________________

Q11 Wireframe: Goal Setting Slides **Section 3. Review Goal Setting and Planning**When someone first starts using Convoy-Pal, we need to know more about the individual, their convoy, and aspects of health and activity that are important to them. Here are a few screen shots to show you what Convoy-Pal’s goal setting section looks like.

Q12 Questions:Do you have any specific input on the goal setting section?

________________________________________________________________

________________________________________________________________

________________________________________________________________

________________________________________________________________

________________________________________________________________

Q13 Wireframe: Image of blood pressure and weight entry with daily vital report. **Section 4. Review Monitoring Options**Once Convoy-Pal knows more about the users, Convoy-Pal can provide tools for monitoring and supporting these goals and overall health. Here you have the option to track things like weight, blood pressure, sleep, medication taking, and nutrition and hydration. Let’s say you would like to check your blood pressure, weight, and temperature daily. Here is how you can do that. Wireframe: medication reminder screen shot.
Convoy Pal also has medication reminders.

Q14 Questions:Do these seem like monitoring tools that would be helpful for people with heart failure and other chronic conditions?

________________________________________________________________

________________________________________________________________

________________________________________________________________

________________________________________________________________

________________________________________________________________

Q15 ****Live Display:**** *Launch “running man” of morning check-in and circle call. Use personal cell phone so they can see that Convoy-Pal will call out.*   **Section 5. Daily Check-In and Calling Circle** Sometimes it is helpful to just a have a quick check-in and a reminder of health-related activities. Here is the daily check-in features. If for some reason, the individual feels they need to contact someone in their Convoy they can do that by using the Calling Circle.

Q16 Questions:What do you think about the daily check-in and calling feature?

________________________________________________________________

________________________________________________________________

________________________________________________________________

________________________________________________________________

________________________________________________________________

Q17 Wireframes: Mobile App and WellAssist Portal **Section 6. Portal and Mobile Application for Convoy**Convoy-Pal also has specific features for the convoy. Family members and friends that have been selected to join the convoy can access information via the website portal and/or the mobile application. On the portal and app, you can see reports about monitoring and send video messages. Here is an image for the main mobile application. Here is also an example of what reports from monitoring and use of the watch look like.

Q18 Questions:What do you think about the portal and mobile app?

________________________________________________________________

________________________________________________________________

________________________________________________________________

________________________________________________________________

________________________________________________________________

Q19 Wireframe: Assessments
**Section 7. Convoy Assessments**Since Convoy-Pal is based on a palliative care approach, Convoy-Pal will also assess psychological, social, and spiritual aspects of health. We ask everyone in the convoy about these aspects. Here is an image of what these assessments/questionnaires look like.*If needed, explain: these aspects related to your mental wellbeing, like sadness, worry, and grief; or family relations and communication; or your thoughts about spiritual or religious wellbeing.* *For providers, they may want to know all the assessments: Edmonton Symptom Assessment Scale (ESAS), FACIT-Pal (Physical, Social, and Emotional), FACIT-SP (Spiritual), Anticipatory Grief Scale (AGS-13), Readiness for ACP (4 item), FACIT-TS-PS (satisfaction with treatment and care).*

Q20 Questions:What do you think about assessing the convoy?

________________________________________________________________

________________________________________________________________

________________________________________________________________

________________________________________________________________

________________________________________________________________

Q21 Are we capturing everything that is important? Probe: She would add anything?

________________________________________________________________

________________________________________________________________

________________________________________________________________

________________________________________________________________

________________________________________________________________

Q22 Wireframes: Use wireframes and links to Resource Library to discuss features and content—Review 1-2 sections based on time.   **Section 8. Convoy Resources** Based on individual **and** convoy responses to the assessments and personally selected goals, Convoy-Pal recommends various tools and resources. Here you see an example of notifications that would connect users to recommendations.   ***Note: Make sure your primary browser is logged-in to Resource Library before interview so links will connect without additional log-ins during interview.***   7a. Structure of Care             Understanding Serious Illness and Palliative Care             Preparing for Clinical Visits

Q23 Question:What do you like and dislike about this section? Any recommendations for improvement?

________________________________________________________________

________________________________________________________________

________________________________________________________________

________________________________________________________________

________________________________________________________________

Q24 7b. Physical Aspects of Care            Tips for Monitoring            Physical Activity, Yoga, Tai Chi

Q25 Question:What do you like and dislike about this section? Any recommendations for improvement?

________________________________________________________________

________________________________________________________________

________________________________________________________________

________________________________________________________________

________________________________________________________________

Q26 Wireframe: Pandora    7c. Psychological Aspects of Care             Music             Art             Mindful Activity

Q27 Question:What do you like and dislike about this section? Any recommendations for improvement?

________________________________________________________________

________________________________________________________________

________________________________________________________________

________________________________________________________________

________________________________________________________________

Q33 Wireframe: MemoryWell    7d. Spiritual Aspects of Care             Life Review and Legacy Tools

Q34 Question:What do you like and dislike about this section? Any recommendations for improvement?

________________________________________________________________

________________________________________________________________

________________________________________________________________

________________________________________________________________

________________________________________________________________

Q28 7e. Nearing the End-of-Life Aspects of CareGrief While Caregiving/Anticipatory GriefLegacy Tools (In addition to Memory Well)Journal

Q29 Question:What do you like and dislike about this section? Any recommendations for improvement?

________________________________________________________________

________________________________________________________________

________________________________________________________________

________________________________________________________________

________________________________________________________________

Q30 7f. Ethical and Legal Aspects of Care            Advance Care Planning            Password Help

Q31 Question:What do you like and dislike about this section? Any recommendations for improvement?

________________________________________________________________

________________________________________________________________

________________________________________________________________

________________________________________________________________

________________________________________________________________

Q32 **Section 9. uMARS**Now that we have reviewed all the Convoy-Pal features and tools. We would like to ask you some specific questions. Feel free to provide additional input as we go along. See Multimedia Appendix 1 PDF: https://mhealth.jmir.org/2016/2/e72/

End of Block: Default Question Block
