# Supplementary material for: Usability and Acceptability of a Palliative Care Mobile Intervention for Older Adults With Heart Failure and Caregivers: Observational Study
Source: JMIR Aging. 2022 Oct 6;5(4):e35592. doi: 10.2196/35592 (PMC9585449; doi:10.2196/35592)
Supplement: Multimedia Appendix 2 [file aging_v5i4e35592_app2.docx]

**Multimedia Appendix 2.** Mean, standard deviation, minimum and maximum values for the scales and subscales of the Mobile Application Rating Scale: User Version (uMARS)

|  | Patients | | | | Caregivers | | | |
| --- | --- | --- | --- | --- | --- | --- | --- | --- |
| Subscales | Mean | SD | Min | Max | Mean | SD | Min | Max |
| Total | 4.00 | 0.78 | - | - | 3.92 | 0.83 | - | - |
| **Section A-Engagement** | **3.80** | **0.79** | **2.25** | **5** | **3.77** | **0.96** | **2.4** | **5** |
| Entertainment | 3.38 | 0.87 | 2 | 5 | 3.33 | 1.63 | 1 | 5 |
| Interest | 3.79 | 1.19 | 1 | 5 | 3.33 | 1.03 | 2 | 5 |
| Customisation | 4.09 | 0.83 | 3 | 5 | 3.80 | 1.10 | 2 | 5 |
| Interactivity | 3.69 | 1.18 | 1 | 5 | 4.17 | 0.75 | 3 | 5 |
| Target group | 4.14 | 0.66 | 3 | 5 | 4.00 | 0.89 | 3 | 5 |
| **Section B-Functionality** | **3.87** | **0.85** | **2** | **5** | **3.82** | **1.00** | **2.5** | **5** |
| Performance | 3.60 | 1.52 | 1 | 5 | 4.00 | 0 | 4 | 4 |
| Ease of use | 4.08 | 0.67 | 3 | 5 | 3.83 | 0.98 | 3 | 5 |
| Navigation | 3.60 | 1.07 | 2 | 5 | 3.20 | 1.10 | 2 | 5 |
| Gestural design | 3.37 | 1.06 | 2 | 5 | 4.00 | 1.00 | 3 | 5 |
| **Section C- Aesthetics** | **4.13** | **0.73** | **3.33** | **5** | **3.89** | **0.72** | **3** | **5** |
| Layout | 3.92 | 0.90 | 3 | 5 | 4.00 | 0.89 | 3 | 5 |
| Graphics | 4.08 | 0.86 | 2 | 5 | 4.00 | 0.63 | 3 | 5 |
| Visual appeal | 4.31 | 0.63 | 3 | 5 | 3.67 | 1.03 | 2 | 5 |
| **Section D-Information** | **4.22** | **0.75** | **2.50** | **5** | **4.21** | **0.64** | **3.5** | **5** |
| Quality of information | 3.92 | 0.90 | 2 | 5 | 3.83 | 0.75 | 3 | 5 |
| Quantity of information | 4.31 | 0.85 | 3 | 5 | 4.33 | 0.52 | 4 | 5 |
| Visual information | 4.42 | 0.67 | 3 | 5 | 4.60 | 0.55 | 4 | 5 |
| Credibility of source | 4.50 | 0.71 | 3 | 5 | 4.25 | 0.96 | 3 | 5 |
